# Optimizing the mirror illusion during mirror therapy: evidence from unimpaired individuals

**DOI:** 10.3389/fpsyg.2025.1666002

**Published:** 2025-10-31

**Authors:** Jin Min Kim, Freya O. Challis, Carmen C. Y. Koo, Jade-Cheuk L. Leung, Leo H. Lo, Sang-Hoon Yeo, T. David Punt

**Affiliations:** ^1^School of Sport, Exercise and Rehabilitation Sciences, College of Life and Environmental Sciences, University of Birmingham, Birmingham, United Kingdom; ^2^Department of Mechanical Engineering, School of Engineering, College of Engineering and Physical Sciences, University of Birmingham, Birmingham, United Kingdom

**Keywords:** mirror therapy, upper extremity, crossmodal illusions, multisensory, embodiment, body representation, stroke

## Abstract

**Introduction:**

Mirror therapy has demonstrated functional benefits for patients recovering from hemiparetic stroke, with its effectiveness primarily attributed to the induction of a compelling visual illusion that engages sensorimotor networks. Although previous research has identified various intervention parameters influencing therapeutic outcomes, a comprehensive understanding of their effects on the illusory experience remains limited. This study investigated how four critical parameters—mirror size (large vs. small), object manipulation (present vs. absent), task complexity (simple vs. complex), and movement execution (unilateral vs. bilateral)—modulate the believability of the mirror illusion in neurologically unimpaired individuals.

**Methods:**

Forty healthy participants performed movements under 16 different combinations of these parameters while receiving mirror visual feedback and rated the believability of the reflected hand on an 11-point Likert scale.

**Results:**

Repeated-measures ANOVA revealed that a large mirror consistently enhanced the illusory experience compared to a small mirror. Although bimanual movements generally resulted in higher believability ratings than unimanual movements, this advantage diminished when complex object manipulation tasks were introduced.

**Discussion:**

These findings suggest that the congruency of multisensory information—between visual, proprioceptive, and motor signals—is critical for maximising the strength of the illusory experience. By identifying the optimal conditions for enhancing the mirror illusion in healthy individuals, this study establishes a foundational framework for adapting and refining mirror therapy protocols in clinical populations.

## 1 Introduction

Mirror therapy has been found to be an effective intervention for the rehabilitation of paretic limbs following stroke (Altschuler et al., 1999). The concept of mirror therapy, introduced by Ramachandran et al. (1995), was initially developed to alleviate phantom limb pain by providing visual feedback that influences somatosensory perception. This approach has since been applied to motor recovery in stroke survivors, most commonly targeting the upper limb, and has been shown to improve both motor impairment and functional performance of the affected limb (Thieme et al., 2018).

Typically, a mirror is placed along the midsagittal plane between the arms, encouraging individuals with stroke to perform bilateral movements while observing the reflection of their unaffected limb as if it were the affected one. Depending on the degree of paresis, hand function, or specific rehabilitation goals, the mirror therapy setup has been frequently modified. These modifications have been deemed acceptable, given that the therapeutic effect of mirror therapy primarily depends on maintaining a strong visual illusion rather than on the specific configuration of the setup.

As mirror therapy fundamentally relies on the induction of a compelling visual illusion, the strength of this illusion is critical to its effectiveness. Illusion strength appears to depend heavily on the congruency of sensory information. When sensory inputs from different modalities are congruent, they are mutually reinforcing and facilitate behavioural responses (Alais et al., 2010). In contrast, conflicts between sensory modalities can disrupt the illusion (Wittkopf et al., 2019), with any incongruence with the visual input threatening to undermine the perceived reality of the affected limb (Synofzik et al., 2006).

Mirror therapy can thus be conceptualised as a form of crossmodal illusion, where visual information alters perceptions from other sensory modalities to create a coherent perceptual experience (Bolognini et al., 2015). Supporting this notion, studies with healthy participants have demonstrated that visual information obtained through the mirror illusion can significantly influence proprioceptive (Snijders et al., 2007) or tactile (Katsuyama et al., 2018) sensations from the unseen hand. Through multisensory integration, the presence of a mirror biases perception toward the (visual) illusory input, reinforcing the effectiveness of the intervention (Holmes et al., 2004; Holmes and Spence, 2005).

Despite its widespread clinical use and documented therapeutic benefits for motor function improvement after stroke, two major challenges remain: the precise neurophysiological mechanisms underlying mirror therapy have yet to be fully elucidated, and the variability in mirror therapy protocols, partly arising from the heterogeneity among stroke survivors, has been identified as a factor that may have weakened the strength and consistency of its effectiveness. Notably, the diversity of intervention approaches complicates efforts to isolate the components most essential for achieving optimal therapeutic outcomes.

As noted above, a strong illusory experience is central to mirror therapy’s effects, and although the precise neurophysiological mechanisms remain incompletely understood, this visual illusion is widely believed to stimulate sensorimotor networks. Mirror therapy’s therapeutic effects are widely believed to arise from the visual illusion of movement, where the reflection of the intact limb is perceived as the affected one. This congruent visual feedback is thought to stimulate sensorimotor networks, facilitating the integration and reorganization of motor pathways (Ezendam et al., 2009; Ramachandran and Altschuler, 2009; Thieme et al., 2013).

Emerging evidence suggests that the perceptual strength—or believability—of the mirror illusion is a critical determinant of therapeutic outcomes (Foell et al., 2014; Deconinck et al., 2015). Stronger illusions have been associated with enhanced neural activation, increased patient engagement, and changes in sensorimotor cortical activity and perception. Consistent with these observations, the engagement of both cortical and subcortical motor-related regions during mirror illusion tasks has been reported (Michielsen et al., 2011; Hamzei et al., 2012), further underscoring the central contribution of the mirror illusion to motor recovery. Collectively, these findings highlight the importance of optimizing the perceptual quality of the mirror illusion to maximize clinical benefits.

To better understand which intervention strategies most effectively harness the therapeutic potential of the mirror illusion, Morkisch et al. (2019) conducted a meta-analysis of 32 studies from the systematic review by Thieme et al. (2018) that reported motor impairment and functional outcomes in stroke survivors. They examined three intervention components: mirror size (small vs. large), movement execution (unimanual vs. bimanual), and movement type (object use vs. no object).

The meta-analysis showed that large mirrors—defined as reaching eye level (50 × 40 cm) following Kim et al. (2017) —were more effective than small ones. Larger mirrors likely make the illusion more immersive by more effectively obscuring the hidden limb and enhancing attention to the reflected hand (Ramachandran and Rogers-Ramachandran, 2019); as noted by Morkisch et al. (2017), reflecting more of the limb may further facilitate adaptation.

They also found that unimanual execution, in which only the unaffected arm moves, produced greater improvements in motor function of the affected limb than bimanual execution. This contrasts with the original protocol of Altschuler et al. (1999), which encouraged symmetrical movement of both arms. Although counterintuitive—since it removes direct training of the impaired limb—Morkisch et al. (2019) proposed that bimanual movement may disperse attention and thereby reduce the therapeutic effect.

Finally, effectiveness was lower when movements involved object manipulation: mirror therapy was more effective when movements were performed without objects. Although such object-based tasks are typically recommended for upper-limb rehabilitation (Bravi and Ellen Stoykov, 2007; Veerbeek et al., 2014; da Silva et al., 2020), manipulating an object during mirror feedback (Higgins et al., 2006; Michaelsen et al., 2006; Arya et al., 2015) may shift attention predominantly toward the seen hand, reducing the salience of the illusory hand and weakening the sense of control.

Consistent with this interpretation, Bai et al. (2019) reported that movement-based mirror therapy —simple joint movements without objects such as joint flexion and extension, gripping/releasing and finger tapping—improved motor impairment more than task-based mirror therapy, which required more complex object-manipulation tasks such as transferring cubes, placing pegs in holes and turning over paper cards. However, because the object-based tasks were also considerably more complex, task complexity itself may have contributed to the observed difference.

These findings, together with previous research, prompted us to consider that the mirror therapy parameters identified by Morkisch et al. (2019) as influencing motor function and recovery might be directly linked to the quality of the illusory experience. Building on this idea, the importance of illusion quality has been recognized as a cornerstone of mirror therapy since its inception (Ramachandran et al., 1995). McCabe (2011) further emphasized that fully believing in the existence of the illusory limb may be critical to the success of the intervention. Supporting this notion, Rowe et al. (2019) provided a more intuitive illustration of the illusory experience’s effects. In their study, they examined how task complexity affected the illusory experience, using a “task realism” scale across 25 different bimanual tasks. Their findings revealed that participants rated simple movements without object manipulation as the most realistic. Given the central role of illusion quality, understanding how individuals experience the reflected hand as part of their own bodily experience during mirror therapy—that is, their sense of embodiment—becomes critical.

The contribution of the crossmodal illusion during mirror visual feedback can be indicated through the investigation of the sense of embodiment (Ernst and Bülthoff, 2004; Wittkopf et al., 2019; Ehrsson, 2020). The sense of embodiment reflects the degree to which an individual perceives the reflected hand in the mirror as their own unseen hand. It is typically conceptualized as encompassing three subcomponents: ownership (whether the mirror image appears to be part of one’s body), agency (whether the individual feels they can control the movement of the mirror image), and location (whether the mirror image is perceived as corresponding to the location of the unseen hand) (Longo et al., 2008).

While embodiment is often investigated through direct evaluation of these subcomponents, it can also be explored through simpler perceptual measures such as the sense of realism (Rowe et al., 2019) or the sense of peculiarity (Fink et al., 1999) during mirror visual feedback. Rowe et al. (2019) asked participants to rate how realistic the mirror reflection felt during various mirror therapy tasks using a 10-point Likert scale, while Fink et al. (1999) asked participants to rate the strangeness or peculiarity of their experience on a scale from 0 (not at all peculiar) to 9 (extremely peculiar). Although these intuitive questions effectively capture immediate perceptual impressions, they primarily reflect aspects related to ownership and did not comprehensively assess the broader cognitive components of embodiment, such as agency and spatial location. Thus, while valuable, these measures offer only a partial perspective on the complex illusory experience elicited during mirror therapy.

Beyond simply experiencing the mirror image as one’s own (ownership), the believability of the illusion provides a more intuitive and integrated assessment of embodiment. Believability directly taps into the process of self-identification (Jeannerod and Pacherie, 2004), encompassing not only the recognition of the illusory limb’s movement (Gallagher, 2000), but also the ability to distinguish between self-generated actions and movements observed externally through the mirror (Jeannerod, 2006).

The potential dissociation between perceiving feeling and believing embodiment has already been highlighted in study of the rubber hand illusion (Tamè et al., 2018). In this study, individuals reported feeling as though a rubber hand was their own while simultaneously recognizing that it was not their actual hand. This distinction highlights the necessity of explicitly assessing believability, as relying solely on perceptual feelings may fail to capture the full extent of the embodied experience.

However, we emphasize that the findings from the rubber hand illusion cannot be directly generalized to the context of mirror therapy. While the rubber hand illusion involves a dummy hand presented alongside synchronous tactile stimulation, mirror therapy uses the reflection of the individual’s actual hand. Consequently, the visual information presented during mirror therapy is regarded as inherently more credible and powerful. Given the veridical nature of the visual feedback, the perceptual conditions are more strongly biased toward belief rather than mere feeling.

The strength of the embodied experience is also highly dependent on the congruency between sensory inputs. Greater congruence between predicted and actual sensory states enhances the belief that the reflected hand is one’s own unseen hand, while greater incongruence (deafference) weakens it (Medina et al., 2015; Moore, 2016). Additionally, mismatches between motor intention or command and actual sensory feedback can disrupt the embodiment experience during mirror visual feedback (Jeannerod, 2006). Such sensory mismatches not only affect body representation but may ultimately undermine the therapeutic effects of mirror therapy (Matamala-Gomez et al., 2020).

This study aimed to investigate the believability of the illusory limb experience (sense of embodiment) during movements performed with mirror visual feedback. Specifically, we examined how this believability was modulated by manipulating four parameters previously identified as important in mirror therapy research: mirror size, movement execution, task complexity, and object manipulation (Morkisch et al., 2019). Participants performed movements commonly used in standard mirror therapy routines. Beyond assessing the realism of the mirror image, evaluating the sense of embodiment allowed us to explore whether the reflected hand was genuinely incorporated into the participant’s self-representation. Through this approach, we sought to address critical implications for optimizing mirror therapy interventions and to identify the conditions most conducive to enhancing the therapeutic effects of the mirror illusion.

## 2 Methods

### 2.1 Participants

Forty right-handed (17 male; mean age: 21.2 years) participants from the undergraduate student body at the University of Birmingham volunteered to take part in the study. All participants were unimpaired and were naïve to the purpose of the study. The handedness of the participants was self-reported. The study was approved by the University of Birmingham’s Science, Technology, Engineering and Mathematics Ethical Review Committee (ERN_15–1,573). Participants provided written informed consent prior to taking part. Recruitment and participation took place between 01/11/2022 and 28/02/2023.

### 2.2 Apparatus

**Figure 1:**
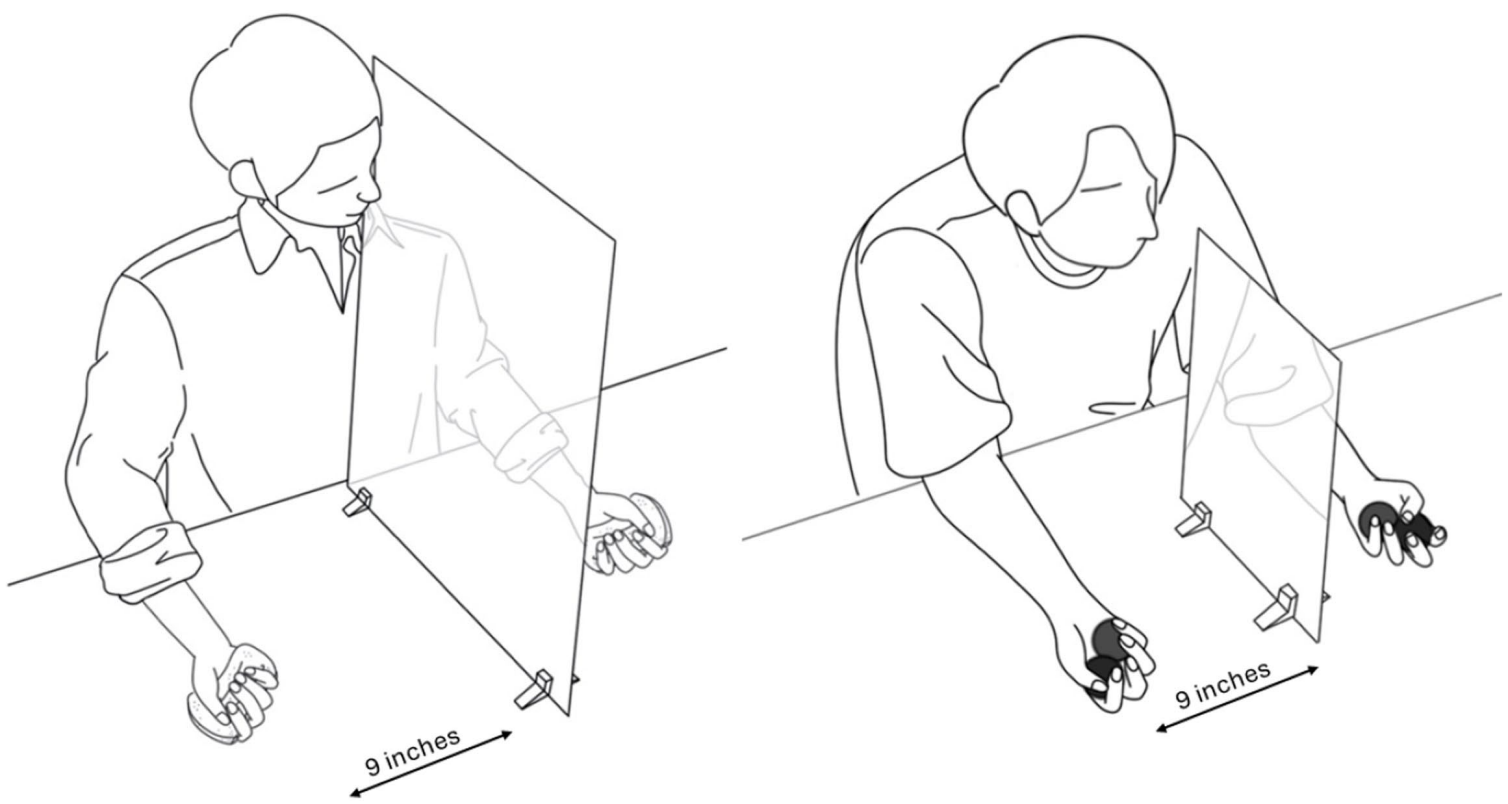
The left figure illustrates one of the conditions in which the participant looks into a large mirror while performing the simple task with manipulating the object. The right figure illustrates one of the conditions in which the participant looks into a small mirror while performing the complex task with manipulating the object. During the trial, participants were directed to look at their hand in the mirror, and both hands were placed 9 inches away from the mirror. Participants completed the task with both hands simultaneously during bimanual execution. However, in unimanual execution conditions, only the hand in front of the mirror (dominant hand) was instructed to move, and the hand behind the mirror did not move with the palm facing up.

The experiment was conducted in the Motor Cognition Laboratory, part of the School of Sport, Exercise, and Rehabilitation Sciences at the University of Birmingham. Two different sizes of landscape-oriented Perspex mirrors (50 cm × 40 cm or 25 cm × 20 cm) were used depending on the conditions. The mirrors were placed perpendicular to the table and aligned to the participant’s mid-sagittal plane using small bespoke wooden mounts. The large and small mirrors were positioned so that the participant’s dominant hand’s reflection was in view—with the center of the mirror and their palm in line. Rather than fixing the eye-to-mirror distance, which would vary depending on individual height and body proportions, the setup was adjusted based on gaze position to ensure that the reflection could be comfortably viewed. The large mirror was fixed with its edge aligned with the table edge, while the small mirror was adjusted to ensure comfort and optimal vision of the reflected limb for each participant. In the small mirror condition, contralateral hand visibility was possible but not controlled, as this was beyond the scope of the study’s purpose. Mirror positions were marked on the table and kept consistent during the experiment. Both hands were placed nine inches away from the mirror to avoid touching the mirror and wooden mounts during the trials (Figure 1).

### 2.3 Task, design and procedure

During the study, each trial required participants to perform self-paced repetitive movements for 20 s. Rowe et al. (2019) implemented a comparable mirror therapy setup in which each task lasted approximately 30 s, including transition time, suggesting our duration falls within a realistic range. Additionally, Kim et al. (2024) reported that the illusion-related drift of the unseen hand emerged within 15 s, indicating that a 20-s trial provides sufficient time for the illusion to take effect. Participants performed 48 trials during the experiment under 16 conditions; participants completed three trials per condition. We adopted three repetitions per condition to ensure measurement reliability, following the precedent of Fink et al. (1999), who repeated each condition three times in a comparable paradigm, whereas Rowe et al. (2019) assessed only a single 20-s trial.

To minimise potential order effects, the 16 conditions were presented in a fully randomised sequence, and each participant was assigned a unique random order. Sixteen conditions were created by a combination of four parameters (Table 1).

**Table 1:** The 16 conditions.

				Movement execution			
				Unimanual execution		Bimanual execution	
				Task complexity			
				Simple task	Complex task	Simple task	Complex task
Mirror size	Large mirror	Object manipulation	Without object	Unimanual simple task without object in large mirror	Unimanual complex task without object in large mirror	Bimanual simple task without object in large mirror	Bimanual complex task without object in large mirror
			With object	Unimanual simple task with object in large mirror	Unimanual complex task with object in large mirror	Bimanual simple task with object in large mirror	Bimanual complex task with object in large mirror
	Small mirror		Without object	Unimanual simple task without object in small mirror	Unimanual complex task without object in small mirror	Bimanual simple task without object in small mirror	Bimanual complex task without object in small mirror
			With object	Unimanual simple task with object in small mirror	Unimanual complex task with object in small mirror	Bimanual simple task with object in small mirror	Bimanual complex task with object in small mirror

The conditions were created by a combination of four parameters: (i) mirror size (large vs. small), (ii) movement execution (unimanual vs. bimanual), (iii) complexity of tasks (simple vs. complex), and (iv) object manipulation (with object vs. without object).

#### 2.3.1 Mirror size (large vs. small)

Large mirror and small mirror were used depending on the condition.

#### 2.3.2 Movement execution (unimanual vs. bimanual)

- Unimanual execution: The task was completed with only the dominant (right) hand in front of the mirror, while the unseen non-dominant (left) hand remained static. While performing unimanual execution and also manipulating an object, the object was not held in the unseen hand, which remained static with the palm facing up.
- Bimanual execution: The hands were instructed to move simultaneously, and while manipulating objects, both hands held objects of the same shape and size.

#### 2.3.3 Complexity of tasks (simple vs. complex) and manipulation of objects (with vs. without object)

**Figure 2:**
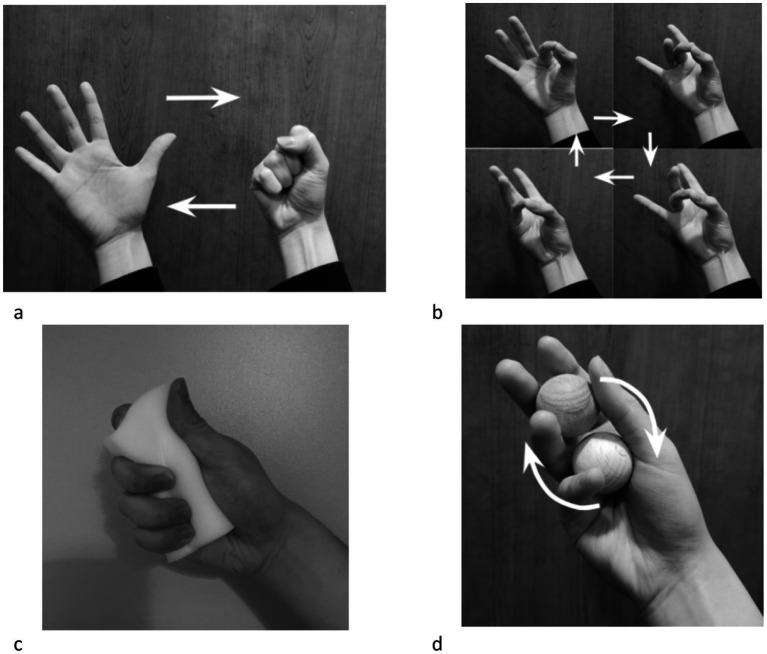
The four conditions combining task complexity and object manipulation parameters. For a ‘simple task without object’, participants were asked to open and close their first repeatedly **(a)**. For ‘complex task without object’, participants were asked to tap their thumb onto the index, middle, ring and little finger in order and repeat **(b)**. For ‘simple task with object’, participants were asked to squeeze the sponge repeatedly with their palms **(c)**. For ‘complex task with object’, participants were asked to rotate two wooden balls either clockwise with the dominant hand and anti-clockwise with the non-dominant hand **(d)**.

- Simple task without object (Figure 2a).
- Complex task without object (Figure 2b).
- Simple task with object: A sponge (9 cm X 4 cm X 2 cm) was given (Figure 2c).
- Complex task with object: Two wooden balls (2.5 cm diameter) were given to each hand (Figure 2d).

In all conditions, participants were instructed to direct their vision to the reflection of the hand in the mirror. Fifteen seconds after the start of each trial, the experimenter asked the participants to rate their believability on a Likert scale with the following question. “How much do you believe the hand in the mirror feels like your left hand?” The question was answered with a number ranging from zero to ten. Zero representing ‘not at all’, whereas 10 represented ‘completely the same’. Once every eight trials, the entire question was posed; the remaining trials only asked for a “please rate from zero to ten” response.

Before commencing the experiment, participants completed the Edinburgh Handedness Inventory and read the written instructions about the procedure of the experiment. Any accessories on the hands and wrists were removed, and any questions regarding the procedure were answered. Participants completed a few practice trials before the experimental trials began in order to familiarize themselves with the procedure. Once experimental trials began, the researcher provided verbal “go” and “stop” signals to indicate the start and finish of trials. Although performance accuracy or precision was not formally scored, the researcher monitored each trial; if the task was not performed adequately (e.g., the ball was dropped during the bimanual ball-rotation task), the trial was immediately stopped and the participant provided their rating. If the performed task lasted less than 5 s, the participant was instructed to repeat the trial within the remaining session. To reduce potential fatigue and to minimise possible carry-over effects, a short rest was provided between trials, with a longer scheduled break implemented after every 20 trials.

### 2.4 Statistical analysis

All statistical analyses were performed using IBM SPSS Statistics (version 29, IBM Corp., Armonk, NY, USA). Data are reported as mean ± SD. Normality of residuals was checked with the Shapiro–Wilk test. A 2 × 2 × 2 × 2 repeated-measures ANOVA was conducted with the within-subject factors Mirror size (large, small), Movement execution (unimanual, bimanual), Task complexity (simple, complex), and Object manipulation (with, without object). Post-hoc pairwise comparisons were Bonferroni-adjusted. Effect sizes are reported as partial η^2^ together with 95% confidence intervals based on the non-central F distribution. The significance threshold was set at α = 0.05.

As a supplementary analysis to verify the robustness and reliability of the believability ratings, we applied linear mixed models (LMM) and cumulative link mixed models (CLMM). The LMM was specified as Believability ~ Mirror size × Movement execution × Task complexity × Object manipulation + (1|Subject) to account for random participant effects in the continuous believability ratings, whereas the CLMM appropriately modelled the ordinal nature of the ratings and provided a non-parametric confirmation of the main effects.

As part of the supplementary analyses, we also assessed trial-to-trial consistency of believability ratings within each of the 16 experimental conditions by computing intraclass correlation coefficients (ICC[2,1]) using a two-way random-effects model with absolute agreement (Shrout and Fleiss, 1979). For each condition, the three trial ratings from every participant were entered as a fully crossed dataset (subjects × trials). The ICC(2,1) quantifies the proportion of total variance attributable to between-subject differences relative to the total variance (between-subject + within-subject + residual error); values approaching 1.0 indicate that believability ratings are highly consistent across the three repetitions of the same condition. 95% confidence intervals for each ICC were computed using an F-distribution approach.

All supplementary analyses—including the LMM, CLMM, and the ICC computations and related visualisations (bar charts, forest plots, participant-level boxplots and spaghetti plots, and the 4 × 4 ICC heatmap)—were carried out using R (version 4.5.1; packages lme4, ordinal, irr, and ggplot2) and MATLAB (R2024b) where appropriate.

## 3 Results

No *a priori* precision target was set. However, with 40 participants, the achieved 95% confidence interval widths for the main effects ranged from approximately ±0.15 to ±0.35 on the 0–10 believability scale, indicating relatively high estimation precision. These values suggest that effects of around 0.5 points could be reliably detected given the observed variability (SD ≈ 2.3). For the key interaction terms, the corresponding 95% CI widths ranged from approximately ±0.20 to ±0.40, indicating comparable precision across main and interaction effects.

### 3.1 Mirror size

**Figure 3:**
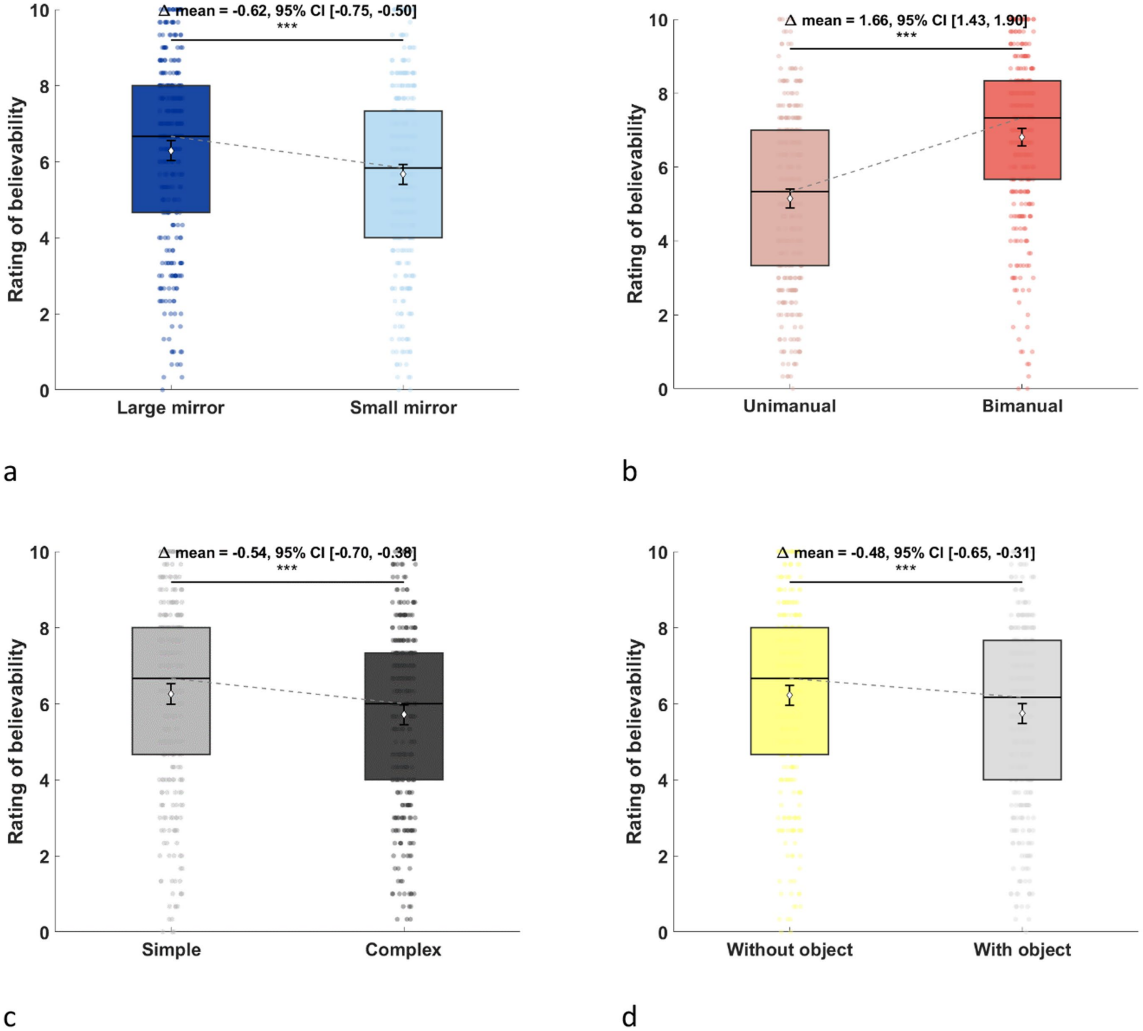
Ratings of believability in each parameter. **(a)** Mirror size. **(b)** Movement execution. **(c)** Task complexity. **(d)** Object manipulation. The effects of each parameter are shown by comparing the two levels. Believability was rated on a 10-point scale, with higher ratings observed for the large mirror, bimanual execution, simple task, and when the object was not manipulated.

The believability was greater when the large mirror (mean = 6.29 ± 2.40) was in place than when replaced by the small mirror (mean = 5.67 ± 2.39), with a mean difference of 0.60 (95% CI [0.45, 0.75]), leading to a significant main effect of Mirror size, *F*(1,39) = 34.23, *p* < 0.001, η^2^_p_ = 0.47, 95% CI [0.28, 1.00] (Figure 3a).

### 3.2 Movement execution

The believability was greater when tasks were performed bimanually (mean = 6.81 ± 2.17) rather than unimanually (mean = 5.15 ± 2.36), with a mean difference of 1.66 (95% CI [1.33, 1.98]), leading to a significant main effect of Movement execution, *F*(1,39) = 37.85, *p* < 0.001, η^2^_p_ = 0.49, 95% CI [0.26, 0.65] (Figure 3b).

### 3.3 Task complexity

The believability was greater when the task was simple (mean = 6.25 ± 2.46) than when the task was complex (mean = 5.71 ± 2.34), with a mean difference of 0.52 (95% CI [0.34, 0.69]), leading to a significant main effect of Task complexity, *F*(1,39) = 18.02, *p* < 0.001, η^2^_p_ = 0.32, 95% CI [0.09, 0.51] (Figure 3c).

### 3.4 Object manipulation

The believability was greater when the tasks were performed without objects (mean = 6.22 ± 2.38) than when performed with objects (mean = 5.74 ± 2.42), with a mean difference of 0.45 (95% CI [0.28, 0.63]), leading to a significant main effect of Object manipulation, *F*(1,39) = 13.31, *p* < 0.001, η^2^_p_ = 0.25, 95% CI [0.06, 0.46] (Figure 3d).

**Figure 4:**
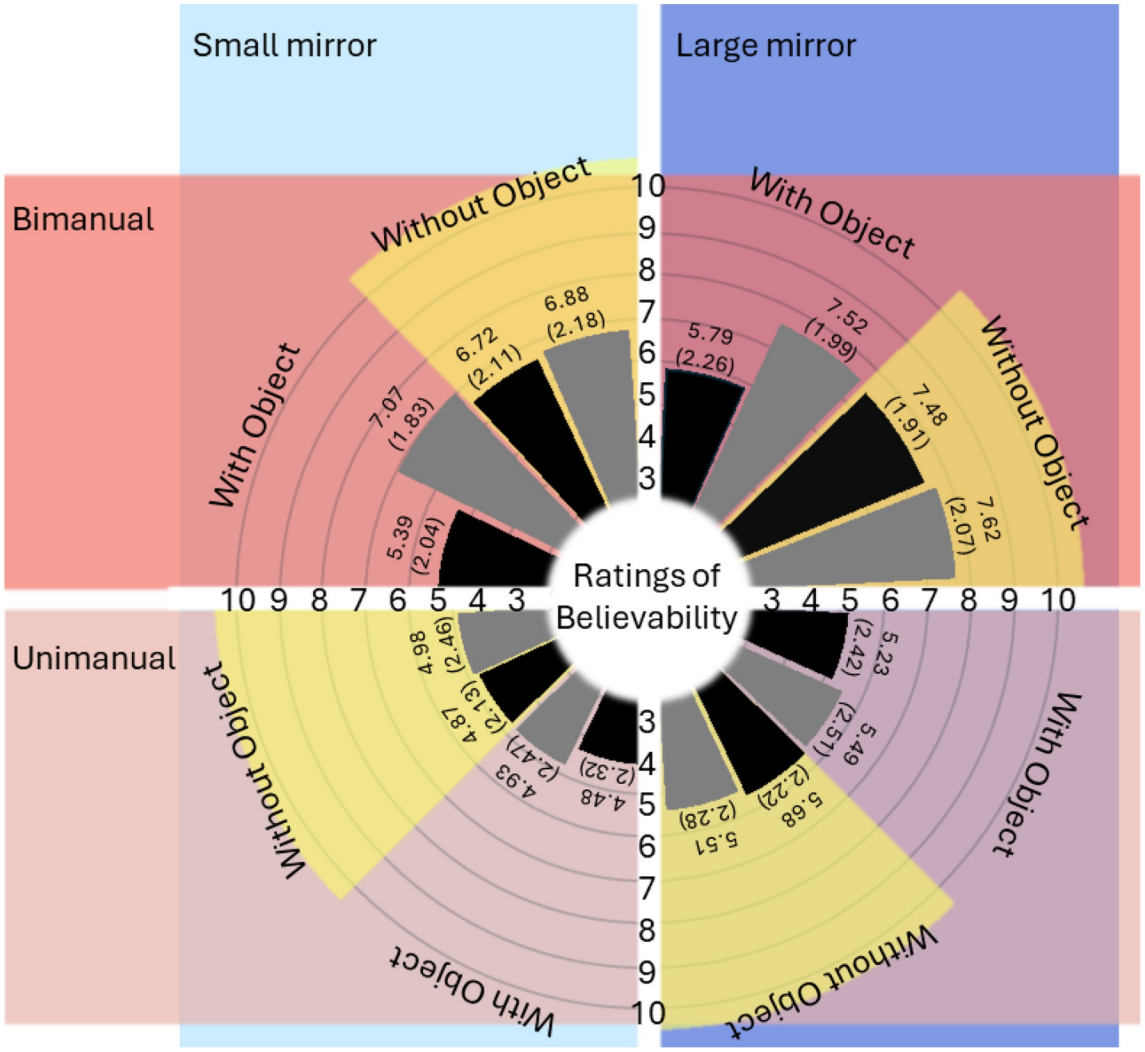
Mean (and standard error) values of believability. The dark blue area represents data collected under the large mirror condition, while the light blue area corresponds to the small mirror condition. The dark red area shows data from bimanual execution, and the light red area displays data from unimanual execution. Four yellow arcs highlight the data collected in the without object condition, distinguishing it from the data related to object manipulation. Black bars represent data from the complex task condition, whereas grey bars depict data from the simple task condition.

**Figure 5:**
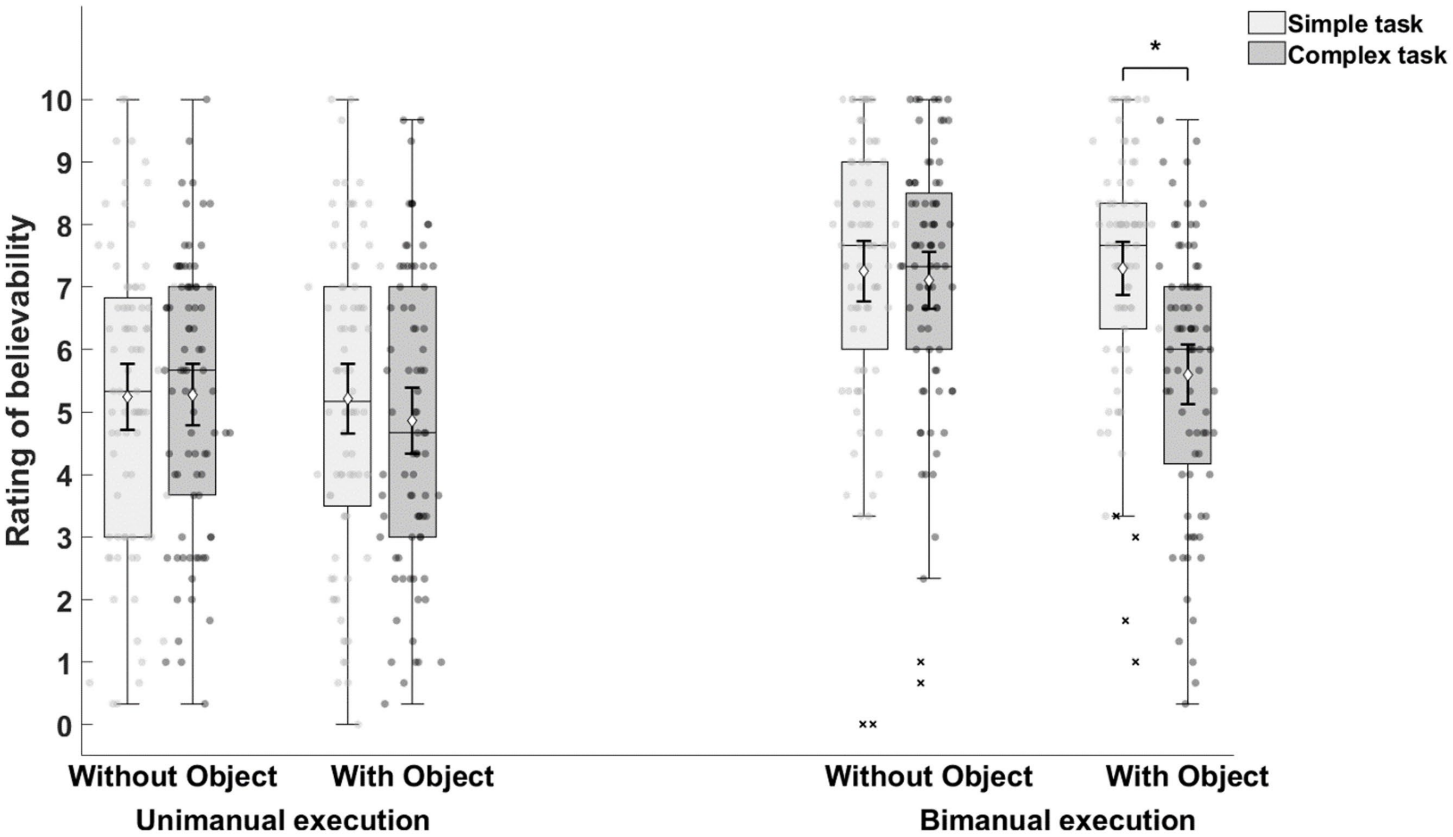
The result of three-way interactions (Movement execution [unimanual, bimanual] × Task complexity [simple, complex] × Object manipulation [with object, without object]). Across the experiment, bimanual execution had higher mean ratings of believability compared to unimanual execution. When tasks were executed unimanually, the mean believability ratings were comparable irrespective of the task complexity and the presence of object used in tasks. However, when tasks were bimanually executed, performing the complex task with object condition significantly decreased the mean ratings of believability.

However, Movement execution x Task complexity, *F*(1,39) = 29.07, *p* < 0.001, η^2^_p_ = 0.43, 95% CI [0.19, 0.60], Movement execution x Object manipulation, *F*(1,39) = 6.78, *p* = 0.01, η^2^_p_ = 0.15, 95% CI [0.01, 0.35], Task complexity x Object manipulation, *F*(1,39) = 36.15, *p* < 0.001, η^2^_p_ = 0.48, 95% CI [0.25, 0.64], and Movement execution x Task complexity × Object manipulation, *F*(1,39) = 11.17, *p* < 0.01, η^2^_p_ = 0.22, 95% CI [0.04, 0.43], interactions suggested a more complex relationship between factors (Figure 4). The results of the three-way interaction are shown in Figure 5.

When performing unimanual movements, the believability between simple and complex tasks with objects, *F*(1,39) = 3.96, *p* = 0.05, η^2^_p_ = 0.09, 95% CI [0, 0.29], and without objects, *F*(1,39) = 0.07, *p* = 0.80, η^2^_p_ < 0.01, 95% CI [0, 0.09], was comparable. However, when performing bimanual movements, object manipulation was responsible for a significant difference between simple and complex tasks, *F*(1,39) = 31.88, *p* < 0.001, η^2^_p_ = 0.45, 95% CI [0.22, 0.62]. Accordingly, bimanual movements that were complex and required object manipulation resulted in significantly lower average believability (mean = 5.60 ± 2.15) than other bimanual execution conditions (mean = 7.22 ± 2.03). Indeed, the lower ratings in this condition were similar to unimanual condition ratings (mean = 5.15 ± 2.36).

## 4 Discussion

Although mirror therapy has been widely shown to improve motor function in the hemiparetic limb following stroke (Thieme et al., 2018), its application across clinical and research settings remains inconsistent. Many studies employ varying protocols without a clear rationale (Morkisch et al., 2017), reflecting the ongoing uncertainty about the most effective therapeutic parameters. Given the heterogeneity among stroke survivors, a uniform intervention strategy is unlikely to be optimal for all individuals (McCabe, 2011), highlighting the need for tailored approaches. Yet, despite increasing clinical use, the identification of condition-specific optimal protocols remains an unresolved challenge.

A recent meta-analysis revealed parameters that resulted in more effective outcomes in hemiparetic stroke survivors (Morkisch et al., 2019). Given that the strength of the illusory experience is thought to underlie the therapeutic effect, we investigated whether these parameters also modulate the illusory experience in unimpaired participants. Consistent with the meta-analysis, it was found that a large mirror elicited a markedly enhanced illusion in comparison to using a small mirror. However, while Morkisch et al. (2019) found unimanual movements were more effective than bimanual movements, we found that bimanual movements were generally responsible for a stronger illusion. Nevertheless, we also found that believability ratings were modified by a combination of factors. More specifically, when unimpaired participants made bimanual movements involving the relatively complex manipulation of objects, the benefits of bimanual movements were lost (i.e., ratings became similar to unimanual movements). Below, these findings are addressed in turn and accounted for, along with what the implications for mirror therapy with stroke might be.

The finding that a large mirror resulted in consistently enhanced believability ratings compared with when a small mirror was in place is consistent with the enhanced effectiveness of mirror therapy demonstrated by the recent meta-analysis (Morkisch et al., 2019). In line with providing what might be considered as a more immersive environment, Rowe et al. (2019) highlights the opportunity afforded by a large mirror to integrate gross muscle movements into a task. This underscores the advantage of a large mirror in reflecting not only the use of the hands, wrists, and forearms but also adequately capturing the movements of the upper arms and shoulders. It allows for the application of tasks that utilize a larger spatial area. McCabe (2011) also highlighted that a large mirror can facilitate a range of bilateral tasks, further expanding its utility. In contrast, a small mirror may limit vision of the illusory limb, and also expose the hidden hand behind the mirror. These factors appear to modulate the quality of the visual illusion created by the mirror and also the effectiveness of mirror therapy.

Several commercially available mirror boxes are small (comparable with the size of the small mirror in this study) offering a limited immersive experience. Instead, the hand is hidden inside the box so that the user may concentrate on their hand in the mirror. However, the enclosed nature of the box may also risk further sensory conflict due to the increased chance of sensory input to the unseen limb (e.g., the hand touching the material that makes up the box sides). Clinical experience suggests patients frequently bump up against the mirror box when moving in limited space available. When this happens, the patient typically pauses the intervention and relocates the position of the unseen hand. This implies that the illusion is disrupted by tactile information gained from touching the box.

In this study, the bimanual execution of movements resulted in generally enhanced believability ratings than those for unimanual movements. As is typical in mirror therapy studies where bimanual movements are employed (Altschuler et al., 1999), participants here made synchronous and symmetrical movements. For unimpaired participants, this clearly results in an experience where one receives visual feedback from the mirror that is congruent with the movements being made with the hidden hand.

It is perhaps not surprising that bimanual movements result in a more believable experience for participants, as they promote congruency across visual, proprioceptive, and motor signals. According to theories of multisensory integration, such congruent input facilitates the construction of a coherent bodily self-representation, thereby enhancing the sense of embodiment (Tsakiris et al., 2010; Kilteni et al., 2016; Ehrsson, 2020). In the context of mirror visual feedback, symmetrical bimanual movements align predicted and actual sensory inputs, reinforcing the embodied experience of the mirrored limb.

Building on this, previous studies with healthy participants have demonstrated that the strength of the mirror illusion can influence not only subjective experience but also motor performance. For example, Holmes et al. (2006) and Snijders et al. (2007) showed that visual feedback from a mirror can bias reaching trajectories and modulate proprioceptive judgments. These findings suggest that enhanced embodiment—via stronger illusions—can directly affect motor behavior, reinforcing the relevance of illusion believability even in non-clinical populations.

Importantly, the clinical significance of this relationship becomes even more evident when considering individuals post-stroke, who often exhibit disruptions in body representation, including impaired limb ownership, altered proprioception, or neglect (Moro et al., 2004; van Stralen et al., 2013). For these patients, mirror therapy may not only support motor recovery, but also aid in recalibrating disturbed body schemas. Enhancing embodiment through congruent and immersive visual feedback could thus serve as a crucial mechanism in restoring both motor and perceptual functions. Accordingly, identifying parameters that reliably increase embodiment—such as mirror size, movement type, and task complexity—may help optimize mirror therapy protocols for this population.

In contrast, unimanual movements uncouple action and perception, producing a mismatch between motor output and the visual feedback provided by the mirror. The findings of Fink et al. (1999) support this explanation: healthy participants reported greater perceptual strangeness(peculiarity) when movement incongruence disrupted the illusion, suggesting that perceptual coherence is key to maintaining embodiment.

While predicted, the finding of a more believable illusion when unimpaired participants made bimanual movements (generally at least) is in contrast with Morkisch et al.’s (2017) finding that unimanual movements (i.e., only moving the unimpaired limb) is a more effective approach than bimanual movements when mirror therapy is applied to individuals with stroke. Of course, the measures here are not the same (illusion believability in this study vs. motor improvement for the meta-analysis by Morkisch et al.), but the contrast remains evident. One might speculate that the relative congruence of the behavioral experience in both cases might explain these distinct findings. Where individuals have unimpaired movement, it seems clear that bimanual movements optimize the illusory experience. However, it was also found that making relatively complex movements and manipulating objects reduced this experience to the level of making unimanual movements. Therefore, perhaps any factor that contributes to a lack of congruence between perception and action (Moore, 2016), even where this might be relatively minor, threatens the illusory experience (Fink et al., 1999). For patients with hemiparesis, it might be hypothesized that making bimanual movements provides no greater sense of congruence than making unilateral movements (Swinnen and Wenderoth, 2004). Further, perhaps making bimanual movements also distracts patients from the therapeutic effects of observing the movement in the mirror. While the results of Morkisch et al.’s meta-analysis are unambiguous, it remains possible that the experience may vary for different patients and understanding these relationships more fully would justify further research.

As noted above, believability ratings in the present study for bimanual movements were modulated by task complexity and object manipulation. In particular, the task of rotating cork balls—previously identified by Rowe et al. (2019) as complex—was rated as difficult by participants in our study. Many participants struggled to coordinate both hands at the same speed and rhythm and occasionally dropped the balls, even during unimanual execution. These difficulties were amplified in the bimanual condition. The attentional demands of the task may have been high enough to exhaust perceptual resources, consistent with Lavie’s (2005) load theory, which posits that under conditions of high perceptual load, the processing of other sensory inputs is suppressed.

However, beyond perceptual load, another plausible explanation for the reduced believability ratings lies in the incongruence of tactile location and proprioceptive feedback between the two hands during object manipulation. Since the ball was not experimenter-controlled, the tactile experience of each hand—where the ball was touched, how it was gripped, and its relative location—likely differed, potentially disrupting the multisensory integration required for a convincing mirror illusion. This mismatch could create a sensory conflict between the visual feedback and the actual somatosensory input, thereby weakening the sense of ownership and agency over the mirrored limb (Longo et al., 2008; Ehrsson, 2020).

Moreover, predicted sensory states, generated through internal forward models (Wolpert and Ghahramani, 2000), may have further contributed to this incongruence. When the visual input from the mirror does not align with the tactile or proprioceptive feedback expected from a motor command, the resulting prediction error can disrupt the sense of agency (Synofzik et al., 2008; Moore, 2016). This conflict is likely to be magnified during tasks that require bilateral coordination under mirrored conditions, where the mapping between efferent commands and afferent sensory signals becomes increasingly ambiguous.

Taken together, while task complexity and cognitive load certainly play a role, sensorimotor incongruence—particularly in tactile location—may be a more decisive factor in undermining the embodied experience during complex bimanual tasks in mirror therapy. Future studies may benefit from controlling for or systematically manipulating tactile congruence to further isolate its effects on illusion strength and believability.

Interestingly, ratings for bimanual execution did not reduce when combined with just one of the other parameters with lower believability (i.e., complexity of task and object manipulation). However, a substantial decline in believability was seen when both parameters were combined, and it appears likely that when ‘overall complexity’ reaches a given threshold it becomes less possible to maintain symmetrical movement of the two limbs and this then threatens the illusion.

As previously discussed, in patients with hemiparesis, even simple movements can be perceived as complex, increasing the risk of perceptual-motor incongruence. In such cases, focusing solely on movements of the unimpaired limb may enhance the believability of the mirror illusion. Our findings indicate that maintaining high perceptual congruency—through reduced task complexity, an appropriately sized mirror, and movement execution tailored to the individual’s motor abilities—can enhance embodiment, potentially supporting motor recovery. While bimanual movements may promote stronger congruency in unimpaired individuals, unimanual execution might be more feasible and effective for stroke patients, especially when motor impairments hinder symmetrical coordination. Additionally, the mirror should be sufficiently large to fully obscure the impaired limb, allowing patients to immerse themselves in the illusory visual feedback. These parameters could serve as guiding principles when designing or adapting mirror therapy protocols to meet diverse patient needs.

While conventional mirror boxes may have limitations in fully supporting these principles, emerging technologies may offer promising alternatives. For example, virtual reality (VR) can create immersive, flexible environments that dynamically adjust the visual representation of limb movements, allowing for greater control over perceptual congruency. Based on our results, VR systems could be programmed to reduce complexity, isolate unimanual input, or scale the visual field to simulate the benefits of a large mirror—thus directly translating our illusion-related findings into applied rehabilitation strategies (Laver et al., 2017; Weber et al., 2019).

Similarly, robotic gloves may enable passive movement of the impaired limb synchronized with the unimpaired limb, producing congruent visual and proprioceptive signals that reinforce the embodied experience. Even though these interventions are technologically advanced, their therapeutic value may ultimately rest on the same foundational principle identified in our study: maintaining perceptual coherence to enhance the illusion of embodiment (Proske and Gandevia, 2012).

A potential direction for future research would be to build upon the current study by incorporating more comprehensive and objective measures, rather than relying solely on brief subjective ratings. It would also be valuable to examine how illusion believability relates to motor performance or brain activation (Song and Kim, 2019), particularly in stroke survivors or in older populations better matched to clinical demographics.

To ensure that our main conclusions were not dependent on the specific analytic approach, we performed supplementary analyses using both a linear mixed model (LMM) (see Supplementary Figure 1) and a cumulative link mixed model (CLMM) (see Supplementary Figure 2). These models, which, respectively, treat believability ratings as continuous and ordinal outcomes, yielded patterns of main and interaction effects consistent with the repeated-measures ANOVA, confirming the robustness of our findings.

Furthermore, to evaluate the consistency of believability ratings across the three repeated trials within each condition (see Supplementary Figures 3, 4), we calculated intraclass correlation coefficients (ICC[2,1]) (see Supplementary Figures 5, 6). ICC values across all 16 experimental conditions ranged from ~0.63 to ~0.83, with most exceeding the commonly accepted threshold for “good” reliability, demonstrating that participants provided highly consistent ratings across repetitions.

Overall, our findings identify mirror size, movement execution, and task complexity as key factors that shape the strength of the mirror illusion and may help guide the development of more effective mirror-therapy protocols. Although we did not perform an *a priori* sample-size or power analysis, we reported effect sizes with 95% confidence intervals, which provides an indication of the robustness of our results. While other limitations—such as the absence of formal movement recordings and the use of healthy adults—remain, these findings offer a strong foundation for future studies that integrate objective motor measures and include clinical populations to translate these illusion-based insights into tailored rehabilitation strategies.

## 5 Conclusion

In summary, we examined the impact of different parameters on the subjective strength of the illusion elicited by mirror therapy in unimpaired individuals, by measuring to what extent participants believed that the hand in the mirror was their unseen hand. Large mirrors elicited higher ratings than small mirrors, and bimanual execution elicited higher ratings than unimanual execution. However, when bimanual execution was combined with a complex task and object manipulation, the believability ratings were markedly lower (comparable with unimanual execution). Overall, findings are consistent with the importance of maintaining congruency between perception and action in order to optimize the illusory experience that is the aim of mirror therapy. Task difficulty threatens this congruence and careful consideration should be paid to the details of the mirror therapy procedure depending in the abilities of individual patients.

## Data Availability

The datasets presented in this study can be found in online repositories. The names of the repository/repositories and accession number(s) can be found at: https://doi.org/10.6084/m9.figshare.29037743.v1.
