# Supplementary material for: Optimizing the mirror illusion during mirror therapy: evidence from unimpaired individuals
Source: Front Psychol. 2025 Oct 31;16:1666002. doi: 10.3389/fpsyg.2025.1666002 (PMC12616857; doi:10.3389/fpsyg.2025.1666002)
Supplement: Supplementary file 1 [file Supplementary_file_1.docx]

Supplementary Material

# Supplementary Figures


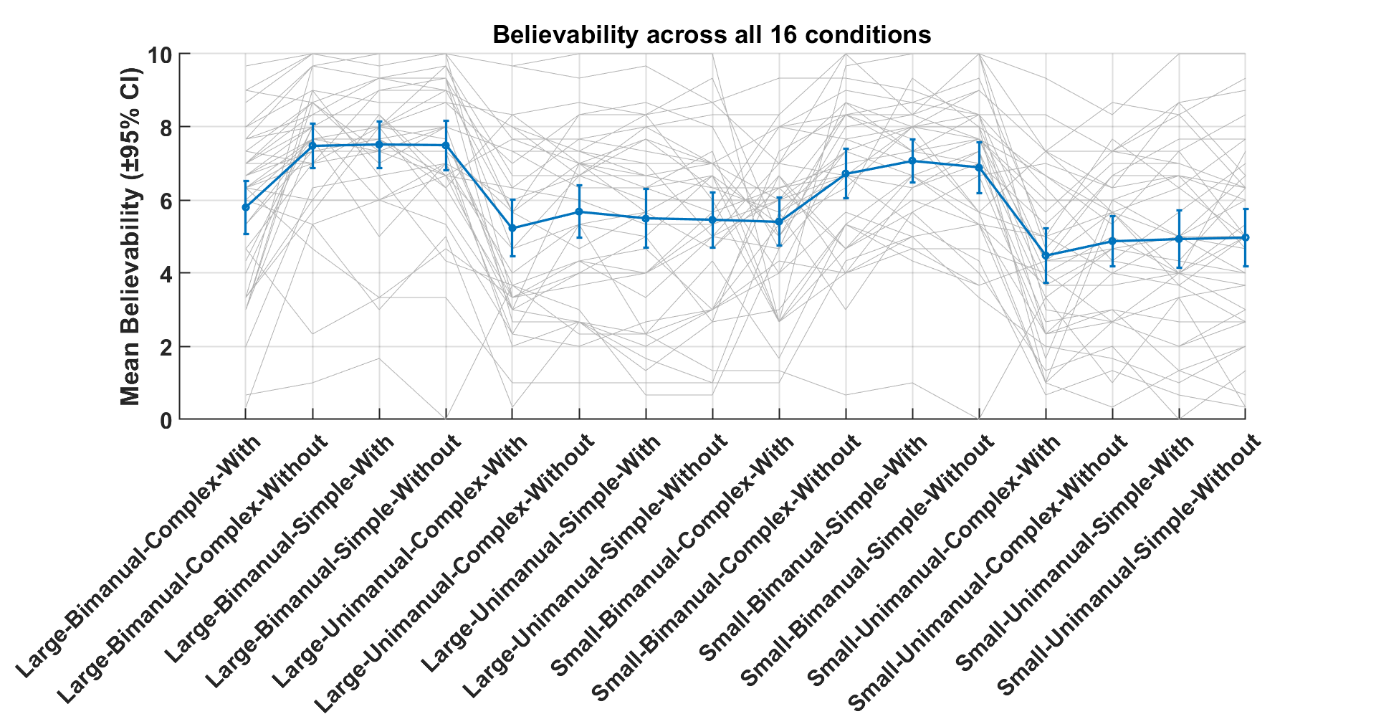


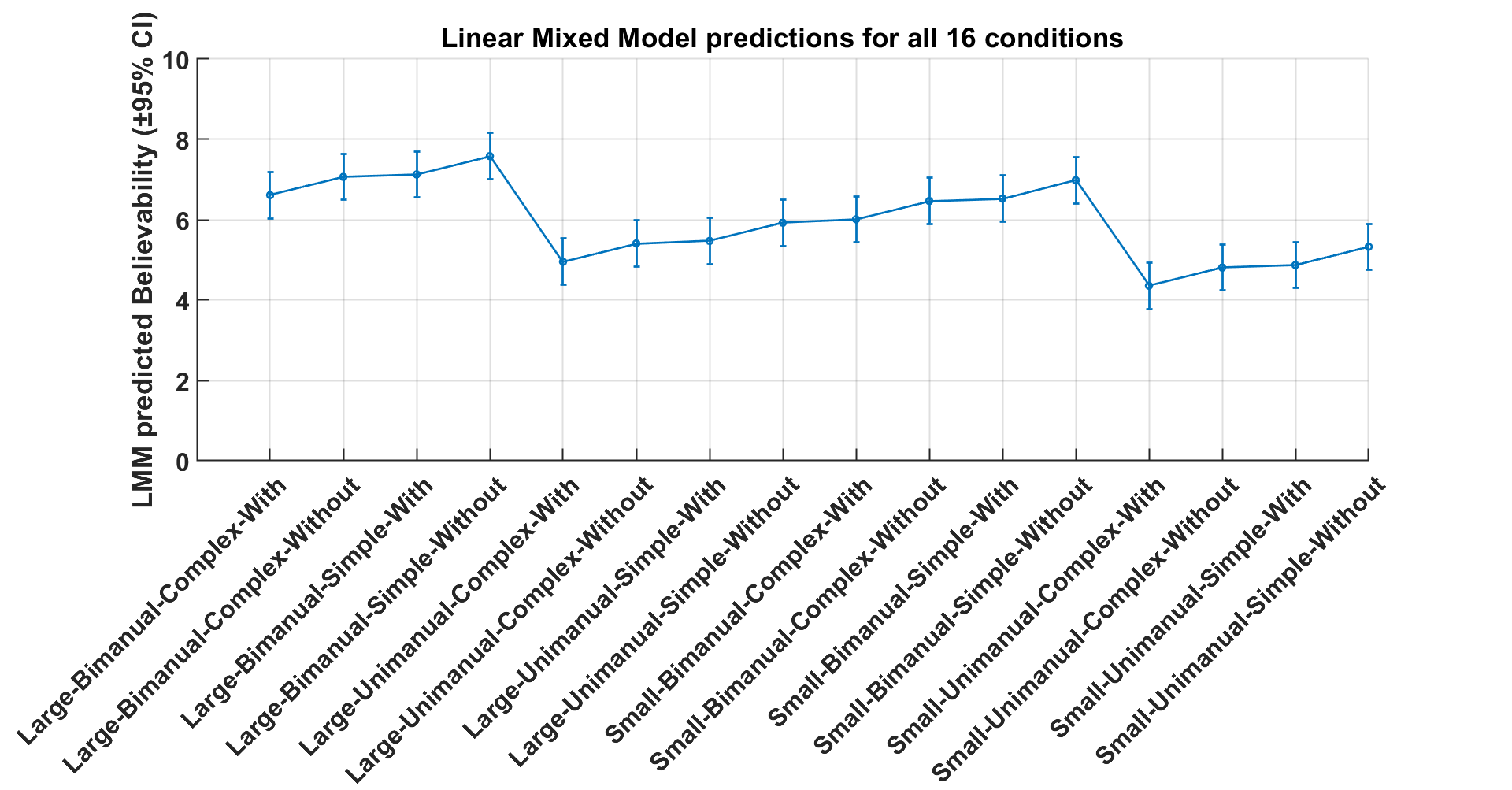


Supplementary Figure 1. Supplementary Figure 1. Mean believability ratings (0–10) for the 16 experimental conditions, shown above as participant means (grey lines, averaged across three trials) with the grand mean (bold coloured line), and below as linear mixed-model (LMM) predictions with bars representing model-estimated marginal means and 95 % confidence intervals. The LMM included Mirror size, Movement execution, Task complexity, and Object manipulation as fixed factors, with a random intercept for each participant.


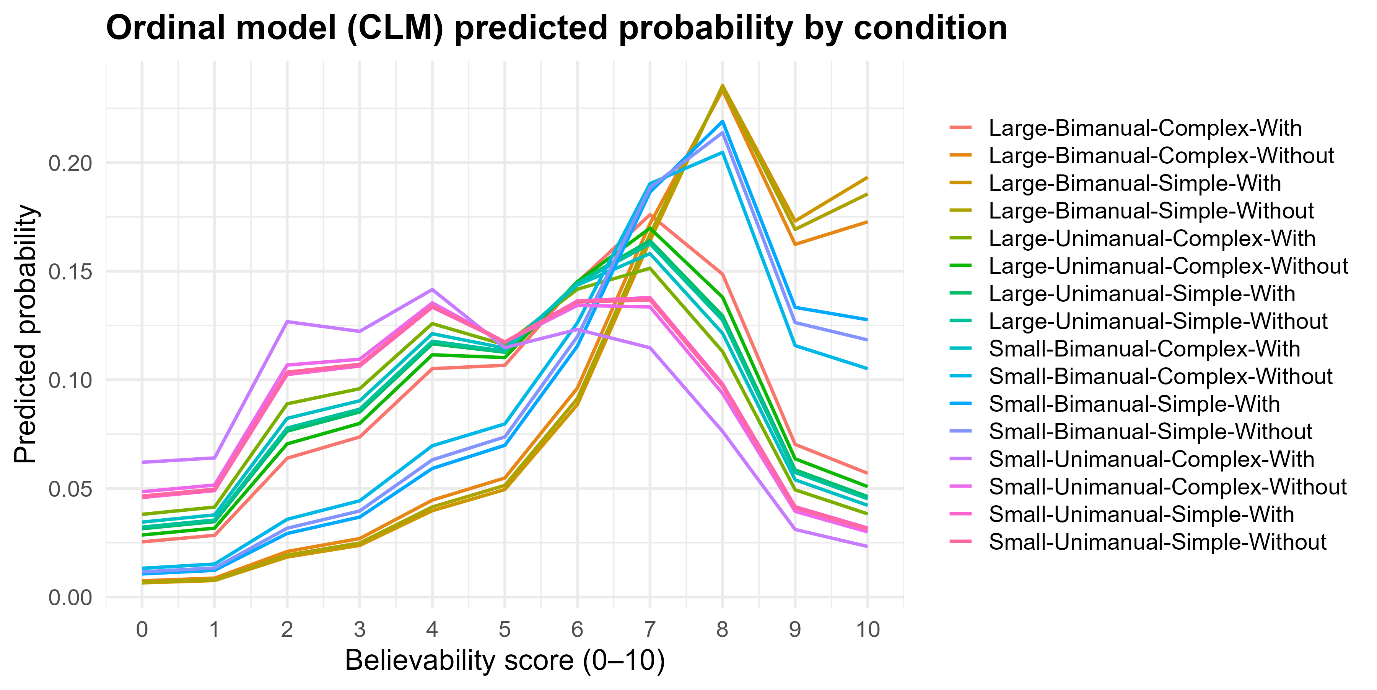

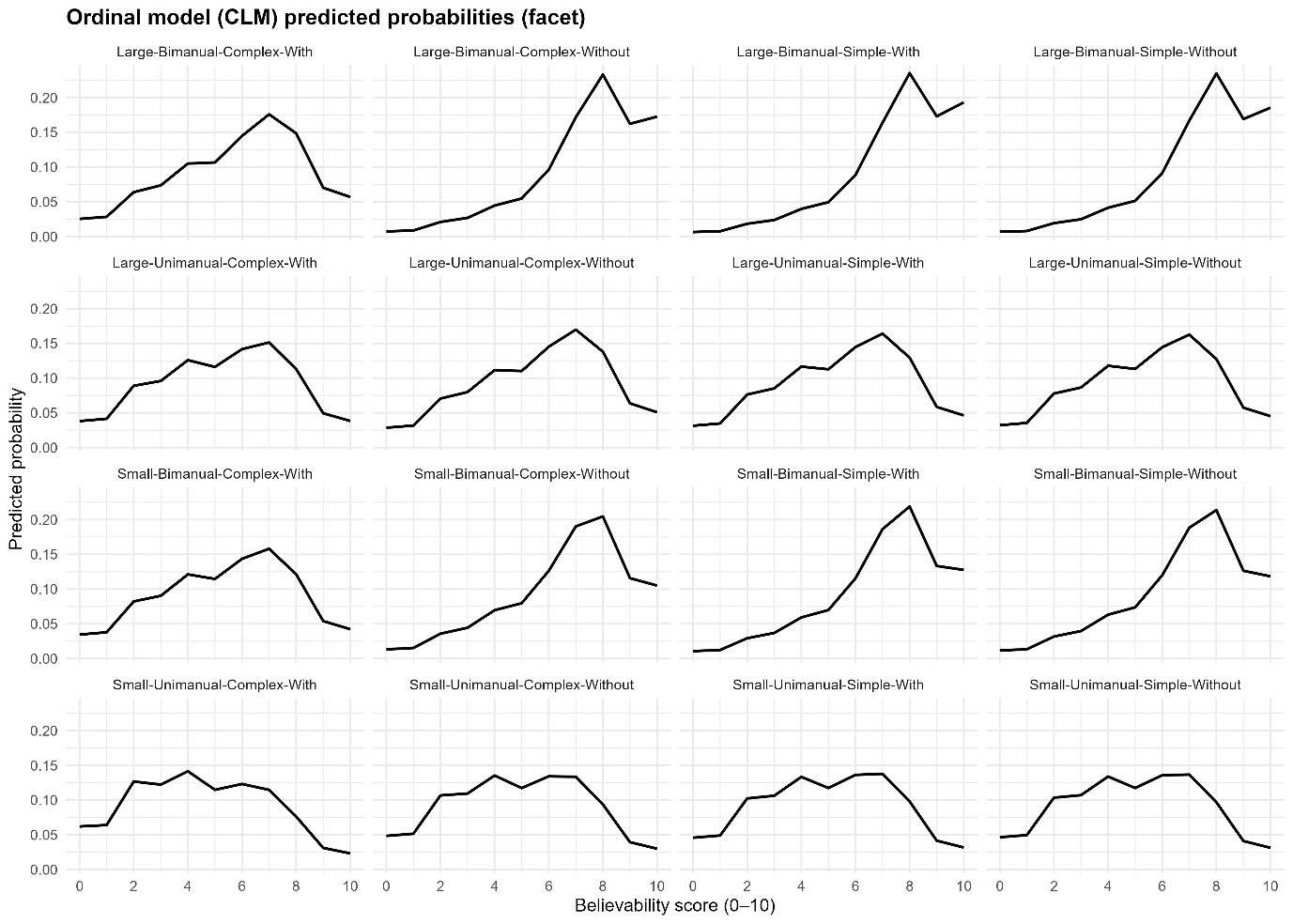


Supplementary Figure 2. Cumulative link mixed model (CLMM) predicted probability distributions of believability ratings (0–10). The upper panel overlays predicted probability curves for all 16 conditions to allow direct comparison of rating distributions. The lower panel shows the same predictions separated by condition to highlight each distribution’s shape. The CLMM treated ratings as ordinal outcomes and included a random intercept for participants.
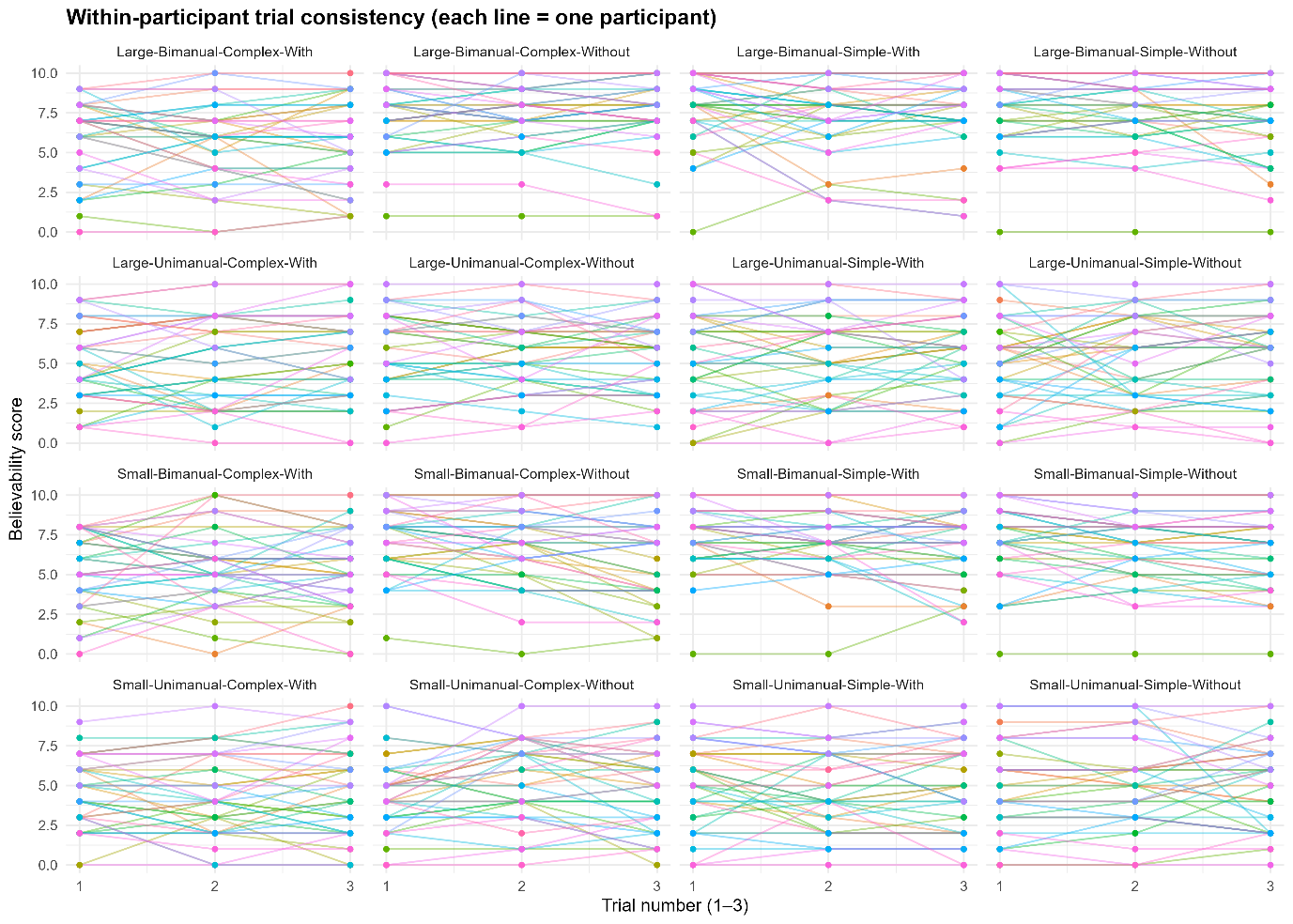


**Supplementary Figure 3.** Within-participant consistency of believability ratings across the three repeated trials in each experimental condition. Each subplot represents one of the 16 conditions (mirror size × movement execution × task complexity × object manipulation). Coloured lines represent individual participants; dots mark their believability ratings (0–10) on each of the three trials. Relatively parallel, horizontally aligned lines indicate that most participants gave similar ratings across trials, consistent with the ICC results*
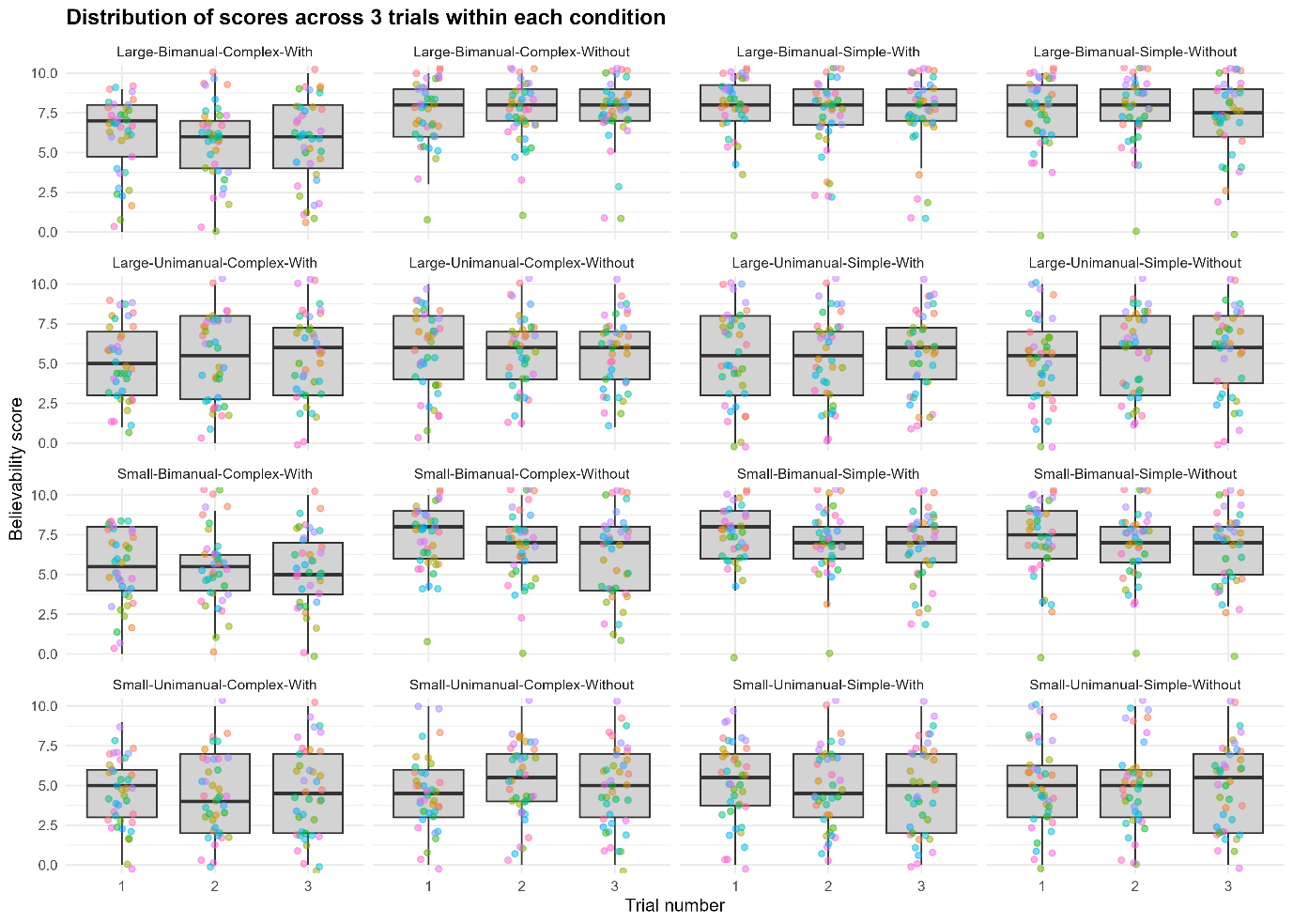
*

**Supplementary Figure 4.** Distribution of believability ratings across the three repeated trials for each of the 16 experimental conditions. Each subplot shows boxplots (median and interquartile range) with overlaid coloured points representing individual participant ratings (0–10) for trials 1–3. Across conditions, the similarity of medians and the compact spread of most boxplots demonstrate that participants tended to give consistent ratings across trials, in line with the overall intraclass correlation coefficients (ICC range ≈ 0.63–0.83; mean ≈ 0.74) indicating moderate-to-good trial-to-trial reliability.


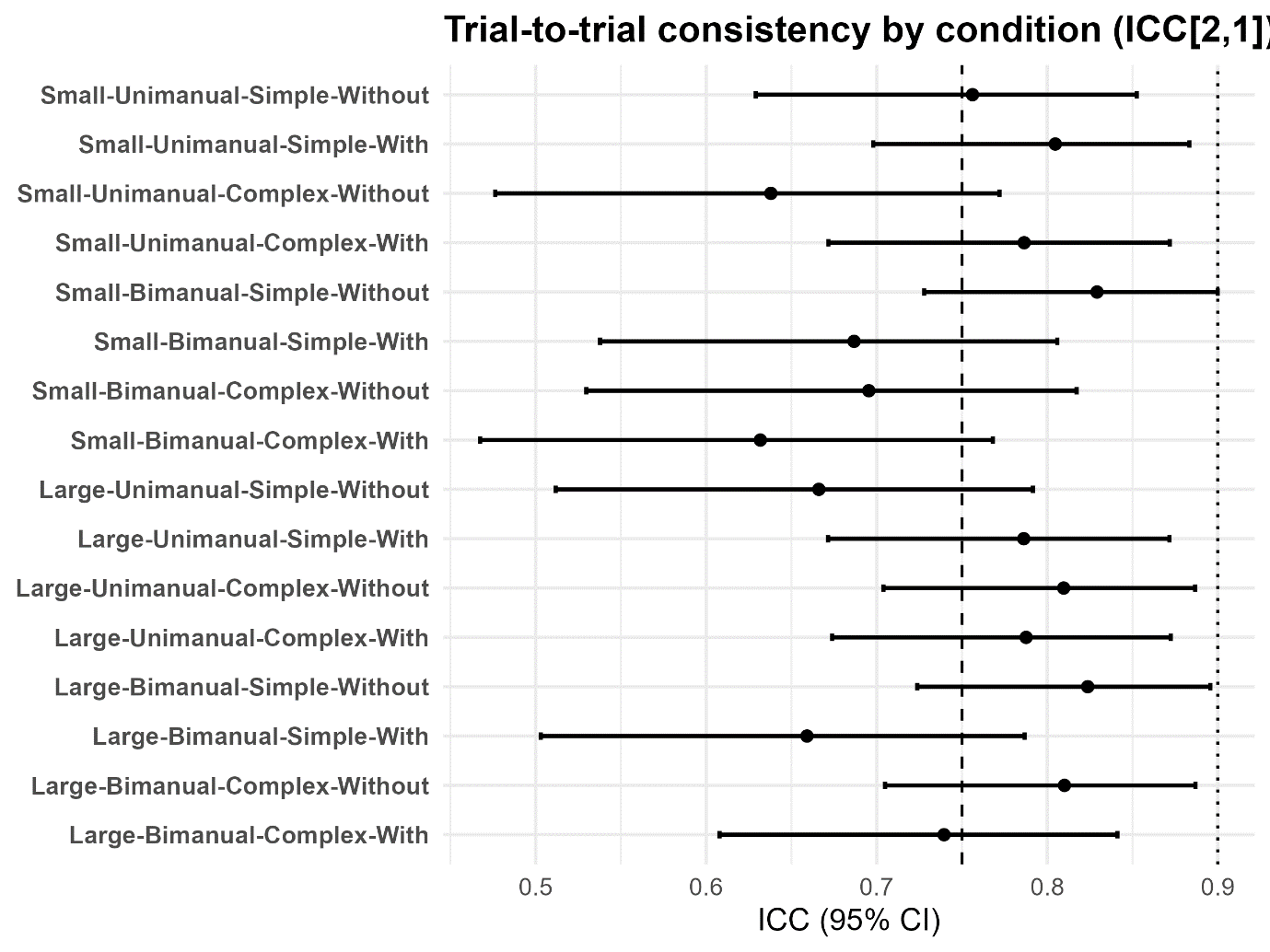
Supplementary Figure 5. Forest plot of intraclass correlation coefficients (ICC[2,1]) with 95 % confidence intervals for trial-to-trial believability ratings in each of the 16 experimental conditions. Dots represent ICC point estimates and horizontal lines show 95 % confidence intervals. The dashed vertical line marks 0.75 (commonly interpreted as good reliability) and the dotted line marks 0.90 (excellent reliability). Higher ICC values indicate greater consistency of participants’ believability ratings across the three repeated trials within each condition.


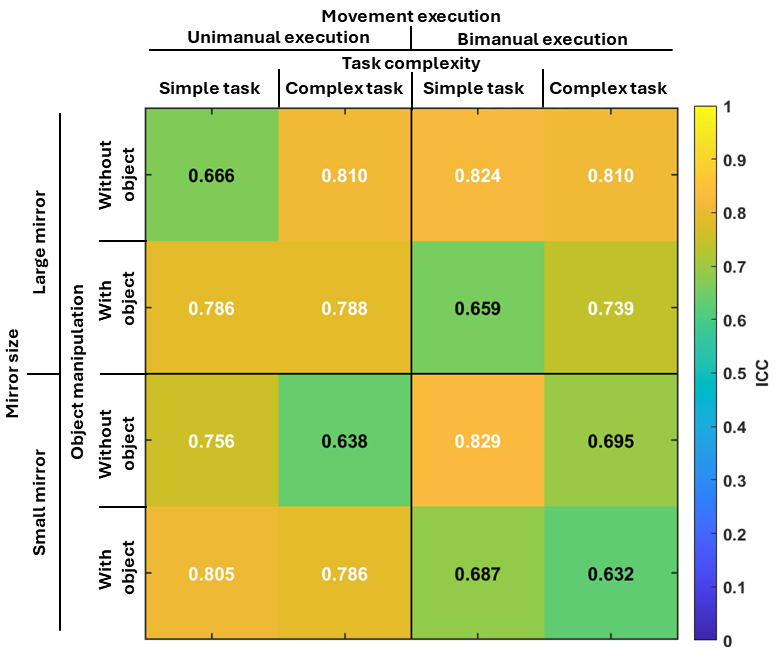


**Supplementary Figure 6.** Heatmap of intraclass correlation coefficients (ICC[2,1]) showing trial-to-trial reliability of believability ratings across the 16 experimental conditions. Cells display ICC point estimates, colour-coded from low (blue/green) to high (yellow) reliability. Columns represent combinations of Movement execution (unimanual, bimanual) and Task complexity (simple, complex); rows represent Mirror size (large, small) crossed with Object manipulation (with, without object). Higher ICC values indicate stronger consistency of participants’ believability ratings across the three repeated trials within each condition. Across all conditions, ICCs ranged from 0.63 to 0.83, with a mean of approximately 0.74, indicating moderate-to-good within-participant reliability of believability ratings.
